# The non-synthetic sweeteners, miraculin and mogroside V, but not stevia, disrupt the intestinal epithelial barrier function through a sweet taste receptor-dependent mechanism

**DOI:** 10.1038/s41598-025-28759-z

**Published:** 2025-12-29

**Authors:** Aparna Shil, Owura Amoakohene, Havovi Chichger

**Affiliations:** 1 Faculty of Biological Sciences, Jahangirnagar University, Savar, Dhaka, 1342 Bangladesh; 2 Anglia Ruskin University School of Life Sciences, Cambridge, CB1 1PT UK

**Keywords:** Intestinal epithelium, Permeability, Natural sweeteners, Sweet taste receptor, Oxidative stress, Caco-2, Biochemistry, Cell biology

## Abstract

Impaired intestinal barrier function is a precursor to various metabolic diseases which can occur when the intestinal epithelium is directly exposed to certain dietary components. Previous studies have demonstrated the barrier disruptive effect of artificial sweeteners such as sucralose and saccharin, in the intestinal epithelium. In the present study, we aimed to evaluate the impact of 3 non-synthetic sweeteners, stevia, miraculin, and mogroside V, on Caco-2 monolayer barrier integrity, reactive oxygen species (ROS) production, and tight junction protein expression as compared to the artificial sweetener saccharin. Miraculin and mogroside V exerted a significant impact on cell numbers with decreased cell viability at both 24 and 48 h. ROS production was also significantly increased by miraculin and mogroside V and epithelial barrier function, assessed by transepithelial electrical resistance and FITC-dextran leak, was significantly disrupted with both miraculin and mogroside V. Stevia exhibited no effect on epithelial cell viability, ROS accumulation or barrier function, despite a range of concentrations investigated. Knockdown of the sweet taste receptor, T1R3, significantly attenuated miraculin- and mogroside V-induced loss of cell viability, ROS accumulation and barrier disruption suggesting a T1R3-dependent mechanism. Gene expression analysis revealed differential regulation of cell junction-related genes by mogroside V and miraculin with upregulated expression of genes such as *CAV1*, *CLDN2* and *CLDN10*, and downregulated expression of genes such as *CAV3* and *CDH2*. These findings demonstrate that miraculin and mogroside V, but not stevia, negatively regulate ROS formation and intestinal epithelial barrier function in a T1R3-dependent manner and potentially through modulation of tight junction proteins. This highlights the need for further investigation into the long-term dietary implications of natural sweeteners, particularly miraculin and mogroside V, on gut health.

**Supplementary Information:**

The online version contains supplementary material available at 10.1038/s41598-025-28759-z.

## Introduction

From an evolutionary perspective, humans have a natural tendency to prefer sweet taste and are inclined to intake sweet foods to increase energy consumption. As such, the use of added sugar in food preparation and processing has become a common phenomenon to make food more palatable and attractive^1^, however, excessive consumption of added sugars in food and beverages has been strongly linked to the global rise in metabolic disorders, including obesity, hypertension, and type 2 diabetes^2,3^. To mitigate the adverse health effects of excessive caloric intake, non-nutritive or less nutritive synthetic sweeteners have been used in the food industry for decades. However, these artificial sweeteners have also become controversial due to their potential negative effects on human health^4,5^ with recent guidelines from the World Health Organization suggesting that artificial sweeteners should not be used as a weight-loss tool due to potential negative impacts on human health and declaring a need for significantly more on the topic^6^.

Of note, the WHO report focused on artificial, and not natural, sweeteners. In more recent years, the family of natural sweeteners, biomacromolecules with potent sweetening abilities, have started to be investigated. This family includes miraculin, mogroside V, and stevia, which are taste-modifying glycoproteins extracted from miracle fruit (*Synsepalum dulcificum* or *Richadella dulcifica*), *stevia rebaudiana* plant, and monk fruit (*Siraitia grosvenorii*) respectively^7,8^. Whilst stevia and mogroside V are between 200 and 400 times sweeter than sucrose, miraculin elicits a response 400,000 times sweeter than sucrose in acidic conditions^7–9^. As such, all 3 natural sweeteners are used as a sweetening agent around the world due to their reduced calorie content, compared to sugar^10^. Mogroside V and stevia are recognised as flavorings by the Flavor and Extract Manufacturers Association (FEMA) and many steviol glycosides derived from stevia, such as rebaudioside A and stevioside, have been granted Generally Recognised As Safe (GRAS) status by FEMA. Despite its frequent use in the food industry, miraculin is not approved as a sweetener by the FDA or the European Food Safety Authority (EFSA). It remains unapproved for use in the United States but has been granted novel food status in the European Union^8^. Although these natural sweeteners are generally considered safe, there are very few research studies performed on them, particularly with regards to the human intestine.

With the recent range of negative findings with artificial sweetener consumption, there is a move towards finding alternative sweeteners which could offer a way to sweeten food without any potential health impacts^11^. In one such study, we demonstrated that the artificial sweeteners, aspartame, saccharin, neotame and sucralose, disrupt intestinal barrier function by inducing epithelial cell death and compromising barrier integrity^12,13^. We have also observed that these sweeteners exacerbate gut microbiota disruption by promoting bacterial pathogenicity^13,14^. A finding mirrored with metagenomic studies into the whole gut microbiome and, alarmingly, indicating that artificial sweeteners can increase glucose intolerance through gut dysbiosis^15^. With the aim of better understanding the effect of natural sweeteners on the gut epithelium, in the present study we employed the widely used immortalized human colorectal adenocarcinoma (Caco-2) cell line as a model for intestinal epithelial cells. We evaluated the impact of natural sweeteners, including the emerging compounds miraculin and mogroside V, as well as the established sweetener stevia, on epithelial cell viability, monolayer permeability, oxidative stress and tight junction gene expression. Given the importance of the sweet taste receptor, T1R3, in regulating the intestinal epithelial response to artificial sweeteners, in the present study we also established whether miraculin, mogroside V and stevia could exert any effect on the epithelium through T1R3. This research provides new insights into how naturally-derived sweeteners could influence gut barrier function and intestinal health. These findings have potential implications for understanding the broader physiological impacts of dietary sweeteners and their contributions to gut health, which are critical in light of increasing consumption trends and dietary shifts.

## Materials and methods

### Cell lines and reagents

Human colon carcinoma cells (Caco-2) were purchased from Sigma-Aldrich (Dorset, UK), cultured in Eagle’s Minimum Essential Media containing 10% foetal bovine serum and 1% penicillin/streptomycin, and used between passages 35 and 50. Caco-2 cells were cultured for 21 days with weekly media change to allow differentiation into an intestinal epithelial model. At day 21, transepithelial electrical resistance (TEER), measured with EVOM2 (World Precision Instruments, Herts, UK), of 300 ohms.cm^2^ or higher was considered to be an intact monolayer. Studies outlined were performed using these differentiated Caco-2 cells. T1R3 and actin antibodies were purchased from Novus Biologicals (Cambridge, UK) using #NLS5060 and #NB600-501 respectively. Silencing RNA (siRNA) and a DharmaFECT™ reagent (T-2001-01) were obtained from Dharmacon (Cambridge, UK) using #D-001810-10-05 for non-specific and #L-022184-00-0010 for T1R3. DCFDA (2′,7′-dichlorofluorescin diacetate) #ab113851 was purchased from Abcam (Cambridge, UK) and the RT^2^ Profiler PCR array for human cell junctions (#PAHS-213Z) was purchased from Qiagen (Manchester, UK). All other reagents, including the artificial sweetener saccharin (#240931), natural sweeteners miraculin (#4016657), mogroside V (#PHL80498), and stevia (#06295001), were purchased from Sigma Aldrich (Dorset, UK).

### Cell viability and oxidative stress studies

Caco-2 cell viability was determined through the MTT (3-(4,5-dimethylthiazol-2-yl)-2,5-diphenyltetrazolium bromide) assay. Cells were treated with saccharin, miraculin, mogroside V, and stevia, at concentrations ranging from 0.0001 to 1000 µM, or with a vehicle control (sterile water) for 24 and 48 h. Following treatment, MTT reagent (5 mg/ml) was added to each well, and cells were incubated for 4 h at 37 °C to allow for formazan crystal formation. Subsequently, 10% sodium dodecyl sulphate solution was added and absorbance was measured at 570 nm using a microplate reader, and cell viability was expressed as %, normalised against the vehicle control.

Caco-2 cells were seeded at a density of 1 × 10^4^ cells per well in a black-walled 96-well plate and incubated for 24 h. The cells were then treated with a cell-permeable, fluorogenic dye, DCFDA, at a concentration of 10 µM, and incubated at 37 °C in the dark for 30 min. Following incubation, DCFDA was removed, and cells were exposed to either 10 µM of miraculin, mogroside V, stevia, and saccharin, or the vehicle control (sterile H₂O) for an additional 1½ h in the presence or absence of N-acetyl cysteine (NAC, 1 mM). Fluorescence intensity of DCFDA was recorded at an excitation wavelength of 488 nm using a fluorescent plate reader (Victor, Perkin Elmer). Results were compared to untreated cells to assess baseline fluorescence levels in the absence of DCFDA.

### Epithelial monolayer permeability and transepithelial electrical resistance

Epithelial monolayer permeability was assessed using the fluorescein isothiocyanate (FITC)-dextran permeability assay and validated with transepithelial electrical resistance. For the analysis of monolayer permeability, Caco-2 cells were plated onto Transwell filters for 24 h, followed by exposure to the sweeteners (10 µM), and the vehicle control (sterile H2O) for a further 24 h. TEER was collected as ohms and presented as ohms x cm^2^ by subtracting the resistance of the blank well (containing no cells) followed by multiplication with the surface area (cm^2^) for the insert used. Where stated, cells were first transfected with siRNA or the vehicle. Permeability was measured by adding FITC-conjugated to 20 kDa dextran (FD20) to media in the upper chamber of the Transwell filter to a concentration of 5 µg/µl. FD20 was allowed to equilibrate for 180 s at 37 ºC, and a sample (100 µl) of media from the lower chamber was collected and analysed at 488 nm using a fluorescent plate reader (Victor, Perkin Elmer). Permeability (%) was calculated by fluorescence accumulated in the lower chamber divided by fluorescence in the upper chamber, which was then multiplied by 100.

### siRNA transfections

Caco-2 cells were transiently transfected with siRNA specific to T1R3 or a non-specific, scrambled control (ns) siRNA using a DharmaFect™ 2 reagent, as previously described (Shil et al. 2020). Cells were transfected at a seeding density of 0.5 × 10^4^ cells per well of a 96-well plate or 2.5 × 10^4^ cells per well of a Transwell insert. At 24 h post-transfection, cells were exposed to sweeteners or vehicle control (H_2_O) for a further 24 h. Experiments were then performed as outlined in “Cell viability and oxidative stress studies” and “Epithelial monolayer permeability and transepithelial electrical resistance”. Western blot analysis was performed to confirm knockdown in T1R3 expression in Caco-2 cells. At 48 h post-transfection, cells were harvested in ice-cold RIPA buffer and cell supernatant was collected following centrifugation as cell lysate. Lysate was loaded onto 10% SDS-PAGE at 50 µg per sample and gel was transferred using a semi-dry protocol. Blots were blocked with 5% milk in TBS-tween for 1 h followed by overnight incubation with T1R3 antibody at 1:1000 concentration ratio.

### Gene expression array studies

Expression of human tight junction-related genes in Caco-2 cells was assessed using the RT^2^ Profiler™ PCR array Human Cell Junction PathwayFinder kit (Qiagen PAHS-213Z). Manufacturer’s instructions were followed for the isolation of RNA from treated Caco-2 cells and the array was run as indicated in guidelines. Data were collected using the Roche Light cycler 480. The second derivative maximum setting was used to obtain C_T_ values from the Light Cycler. An automated baseline was established using the Light Cycler, and the threshold value for all profiler arrays was established within the lower third of the linear phase of amplification plots. Raw C_T_ data were then exported to Excel for further analysis. Data were normalized for mRNA expression to stably-expressed housekeeping genes (*ACTB, B2M*, and *GAPDH*).

### Statistical analysis

Experiments were repeated a minimum of 4 times with sample number presented in the legend for each experiment and all experiments performed in duplicate. Data were analysed using GraphPad Prism 7.0. For three or more groups, variance was assessed using Bartlett’s test. For datasets which did not meet the assumption of normality or heterogeneity of variance, differences between groups were analysed using the Kruskal–Wallis test followed by Dunn’s post-hoc test. For all other data sets, differences among the means were tested for significance in all experiments by ANOVA with Tukey’s range significance difference test. Significance was reached when p < 0.05. Values are presented as mean ± standard error mean (S.E.M.).

## Results

### Miraculin and mogroside V, but not stevia, elevate oxidative stress to cause cell death in Caco-2 cells

**Fig. 1:**
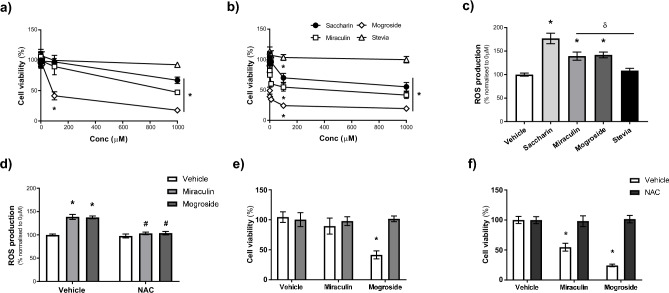
Miraculin and mogroside V, but not stevia, elevate oxidative stress to cause cell death in Caco-2 cells. Panels (**a**–**f**) Caco-2 cell viability was assessed using the MTT assay following 24 h (**a** and **e**) and 48 h (**b** and **f**) exposure to sweeteners miraculin, mogroside V, stevia and saccharin at concentrations from 0.0001 µM to 1000 µM, or the vehicle control (sterile H₂O), in the presence (**e** and **f)** or absence (**a** and **b**) of NAC (1 mM). Panels (**c** and **d**) *ROS production was measured using* DCFDA (10 µM) pre-incubation followed by Caco-2 cell exposure to miraculin, mogroside V, stevia, and saccharin (10 µM) or the vehicle control (sterile H₂O) for an additional 1½ h in the presence (**d**) or absence (**c**) of NAC (1 mM). Data are presented as mean ± S.E.M. n = 5–6. *p < 0.05 versus vehicle control for sweeteners, ^δ^p < 0.05 versus saccharin treatment, ^#^p < 0.05 versus vehicle control for NAC.

Considering the varying levels at which different sweeteners are consumed in diets, and building on our earlier research on artificial sweeteners, we aimed to investigate how the viability of Caco-2 cells is influenced by different physiologically-achievable doses of the natural sweeteners, miraculin, mogroside V, and stevia, as compared to the artificial sweetener, saccharin. We investigated the concentration range 0.0001 µM to 1 mM for sweeteners at both 24 h and 48 h (Fig. 1a and b respectively). As previously shown (Shil et al., 2020), saccharin resulted in a significant decrease in Caco-2 cell viability at 1 mM at 24 h (Fig. 1a) and at both 100 µM and 1 mM at 48 h (Fig. 1b). Miraculin exerted a similar effect on Caco-2 cell viability at both 24 and 48 h with significant loss of viability at the same concentrations as saccharin. In contrast, mogroside V caused a significant reduction in cell viability at 100 µM at 24 h, and a more dramatic drop at both 24 and 48 h compared to saccharin and miraculin (Fig. 1a and b). Stevia, by comparison, had no impact on Caco-2 cell viability at any concentration or timepoint investigated (Fig. 1a and b). Our previous studies have demonstrated that artificial sweetener-induced Caco-2 cell death is ROS-dependent^12^ therefore we next assessed the impact of natural sweeteners mogroside V, miraculin and stevia on oxidative stress. To ensure findings were not caused by Caco-2 cell death, we used a sub-lethal dose of sweeteners (10 µM). As previously shown, saccharin significantly elevated ROS accumulation and, whilst miraculin and mogroside V exposure caused significant elevation in ROS levels, this was significantly lower than saccharin-induced oxidative stress (Fig. 1c). In contrast, stevia treatment had no impact on Caco-2 cell ROS accumulation (Fig. 1c). Interestingly, in the presence of the antioxidant, NAC, miraculin and mogroside V were not able to exert the increased ROS effects on Caco-2 cells (Fig. 1d). Furthermore, NAC exposure attenuated sweetener-induced cytotoxic effects on Caco-2 cells with loss of cell viability induced by 10 µM miraculin or mogroside V blocked by the antioxidant at 24 h (Fig. 1e) and 48 h (Fig. 1f). Overall, these results suggest that, at physiologically-relevant concentrations, the natural sweeteners miraculin and mogroside V cause Caco-2 cell death through a ROS-dependent pathway. Further, these studies demonstrate that, despite also being a natural sweetener, stevia does not impact Caco-2 cell viability or ROS accumulation.

### *Miraculin and mogroside V regulate Caco-2 cell ROS accumulation and loss of viability *via* the sweet taste receptor, T1R3*

**Fig. 2:**
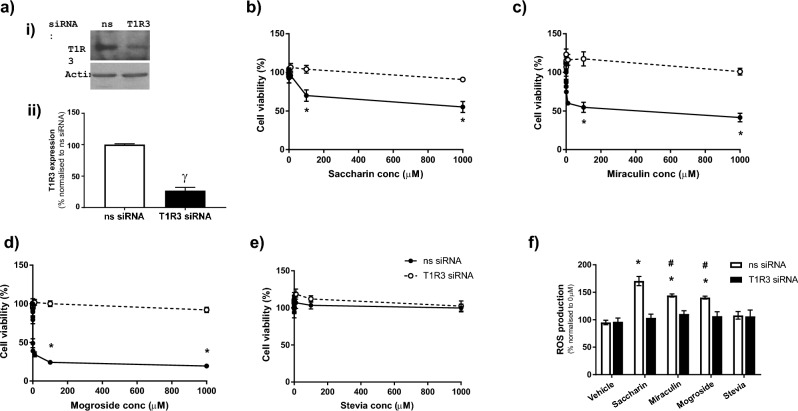
Miraculin and mogroside V regulate Caco-2 cell ROS accumulation and loss of viability via the sweet taste receptor, T1R3. Panel (**a**) siRNA knockdown of T1R3 was performed using specific siRNA or a non-specific (ns) control. Reduction in T1R3 expression was confirmed using Western blot analysis as a representative blot (i) and quantification (ii). Panels (**b**–**e**) Caco-2 cell viability was measured using the MTT assay following knockdown of T1R3 at concentrations from 0.0001 µM to 1000 µM, or the vehicle control (sterile H₂O), of saccharin (**b**), miraculin (**c**), mogroside V (**d**), and stevia (**e**). Panel (**f**) ROS production was measured using DCFDA following knockdown of T1R3 in response to saccharin, miraculin, mogroside V and stevia (10 µM). Data are presented as mean ± S.E.M. n = 5–6. *p < 0.05 versus vehicle control for sweeteners, ^γ^p < 0.05 versus ns siRNA, ^#^p < 0.05 versus saccharin treatment.

Non-synthetic sweeteners, such as miraculin, mogroside V and stevia, act on the T1R2/3 complex to elicit sweet taste sensing^16^. We have previously demonstrated the predominance of T1R3 in the intestinal epithelium^12^ therefore our next studies sought to understand whether miraculin and mogroside V are exerting a cytotoxic effect on Caco-2 cells through T1R3. Following molecular inhibition of T1R3, we observed 73.1 ± 5.8% knockdown (Fig. 2a). As expected saccharin-induced cell death and ROS accumulation was attenuated by T1R3 knockdown (Fig. 2b and f). Interestingly, this same ablation was noted for miraculin and mogroside V-induced cell death and oxidative stress (Fig. 2c,d and f). As would be expected given the absence of impact of stevia, T1R3 knockdown had no impact on cell viability or ROS accumulation in the presence of stevia (Fig. 2e and f). These findings demonstrate that, much like artificial sweeteners, mogroside V and miraculin are interacting with the sweet taste receptor, T1R3, to elicit a cytotoxic and oxidative stress effect on Caco-2 cells.

### At sub-toxic concentrations, miraculin and mogroside V, but not stevia, disrupt epithelial barrier function through T1R3

**Fig. 3:**
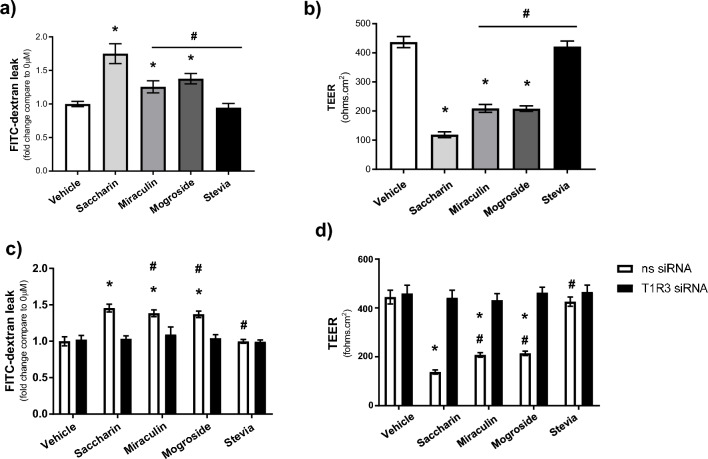
At sub-toxic concentrations, miraculin and mogroside V, but not stevia, disrupt epithelial barrier function through T1R3. Intestinal epithelial barrier function was measured in Transwell arrays using FITC-dextran (**a** and **c**) and TEER (**b** and **d**) following exposure to sweeteners for 24 h at 10 µM (**a** and **b**) and in the presence and absence of T1R3 siRNA (**c** and **d**). Data are presented as mean ± S.E.M. n = 6. *p < 0.05 versus vehicle control for sweeteners, ^#^p < 0.05 versus saccharin treatment.

Given the key role which artificial sweeteners play in regulating epithelial barrier function, we next assessed the effect of non-synthetic sweeteners, miraculin, mogroside V and stevia, on trans-epithelial electrical resistance and FITC-dextran leak. Using sub-toxic concentrations of the sweeteners, 10 µM for 24 h, we found that both miraculin and mogroside V cause a significant increase in leak of FITC-dextran (Fig. 3a) and decrease in TEER (Fig. 3b) across the intact epithelial barrier. Interestingly, this miraculin- and mogroside V-induced leak was significantly lower than the barrier-disruptive effect of saccharin but was completely absent in the presence of stevia (Fig. 3a and b). Following molecular inhibition of T1R3, FITC-dextran leak and drop in TEER induced by miraculin, mogroside V and saccharin was completely attenuated (Fig. 3c and d) demonstrating the key role which T1R3 plays in regulating the epithelial barrier function. As previously noted, with the absence of any impact of stevia on barrier integrity, T1R3 knockdown had no further effect (Fig. 3c and d). These findings indicate that miraculin and mogroside V can disrupt the intestinal epithelial barrier in vitro by acting on T1R3.

### Miraculin and mogroside V, but not stevia, differentially regulate cell junction gene expression

**Fig. 4:**
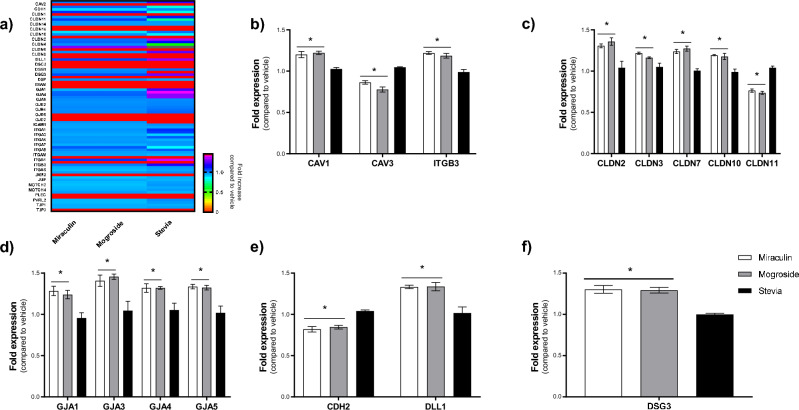
Miraculin and mogroside V, but not stevia, differentially regulate cell junction gene expression. Expression of human tight junction-related genes in Caco-2 cells was assessed using the RT^2^ Profiler™ PCR array Human Cell Junction PathwayFinder kit in cells exposed to miraculin, mogroside V and stevia (10 µM). Panel (**a**) Heat map to demonstrate the changes in expression of tight junction-related genes. Panels (**b**–**f**) Fold expression differences observed by classification; focal adhesions (**b**), tight junctions (**c**), gap junctions (**d**), adherens junctions (4**e**), and desmosomes and hemidesmosomes (**f**). Data are presented as mean ± S.E.M. n = 5. *p < 0.05 versus normalised vehicle fold expression (1).

To understand the molecular mechanism through which miraculin and mogroside V could be affecting the epithelial barrier, our final studies utilised a cell junction gene array to investigate the effect of a sub-toxic concentration (10 µM, 24 h) of the natural sweeteners on Caco-2 cells. Differential expression of a range of cell junction genes was noted with miraculin and mogroside V as compared to stevia (Fig. 4a). Significant changes in gene expression were assessed based on cell junction family type. Along with the tight junctions (Fig. 4c) and gap junctions (Fig. 4d) classification, we also investigated the different types of anchoring junctions class; focal adhesions (Fig. 4b), adherens junctions (Fig. 4e), and desmosomes and hemidesmosomes (Fig. 4f). Miraculin and mogroside V exposure, compared to vehicle treatment, resulted in significant upregulation of tight junction genes *CLDN2, CLDN3, CLDN7, CLDN10* (Fig. 4c), and gap junction genes *GJA1*, *GJA3*, *GJA4* and *GJA5* (Fig. 4d). The anchoring junction genes *CAV1*, *ITGB3, DLL1 and DSG3* were also significantly upregulated (Fig. 4b,e, and f). In addition, the cell junction genes *CAV3*, *CLDN11*, and *CDH2* were significantly downregulated with miraculin and mogroside V exposure compared to vehicle-treated cells (Fig. 4b,c,e and f). In contrast, stevia exposure had no significant impact on the expression of any of these genes as compared to the vehicle treatment (Fig. 4b–f). Taken together, these data demonstrate a significant reorganization of cell junction gene expression following treatment with the natural sweeteners miraculin and mogroside V, which span across the different classes of anchoring junctions.

## Discussion

Natural sweeteners such as stevia, miraculin, and mogroside V have rapidly gained popularity in the global food industry, driven by consumer demand for healthier sugar alternatives^11,17^. Among these, stevia is the most widely adopted, due to its high sweetness intensity, thermal stability, and regulatory approval by FAO/WHO Joint Expert Committee on Food Additives (JECFA) in 2004^11^. Mogroside V has rapidly become the second most consumed natural sweetener, owing to its rich sweetness profile and expanding commercial use^7^. Miraculin, in contrast, functions primarily as a taste-modifying protein rather than a direct sweetener; despite pending FAO approval, it is already applied in the food industry to enhance flavour and alter sourness perception^8^. Despite their widespread consumption, the impact of these compounds on intestinal epithelial function and barrier integrity remains insufficiently understood. Parallel to these natural sweeteners, artificial sweeteners, such as saccharin, continue to be widely consumed. However, concerns about their potential effects on gut health and epithelial integrity have prompted deeper scrutiny of both natural and synthetic alternatives. While global sugar intake in beverages has decreased (1.3 kg/capita, 12.6%), consumption of non-caloric sweeteners has risen (0.2 kg/capita, 18.8%)^18^, making it essential to understand their biological consequences.

In the present study, we investigated the effects of stevia, miraculin, and mogroside V, versus saccharin, on intestinal epithelial cells, focusing on viability, oxidative stress, barrier integrity, and junctional gene expression. To understand the impact of sweeteners on the epithelium, we sought to investigate a range of concentrations which could reflect a dose of each which could represent what the gut epithelium would be exposed to. For example, it has been established that 960 mg/L miraculin is found in the natural source, miracle fruit^19^. Given the molecular weight for the sweetener, that would indicate 38 µM as a relevant concentration. Similar consideration of mogroside V was based on their proposed levels in foods, ranging from 350 to 1000 mg/L which would indicate 272 to 777 µM^20^. This concentration is lower for stevia with a maximum permitted level of 330 mg/L set by the European Union equivalent to 341 µM^21^. Given this wide range of concentrations, and the limited available data related to these sweeteners and the gut epithelium, we investigated the impact of low concentrations which are nearly 1000-fold lower than those expected in foods (0.0001 to 10 µM), and up to very high concentrations which exceed those expected in foods by tenfold (1 mM). Notably, with these different natural sweeteners and different concentrations, we observed divergent outcomes. At the shorter time-point (24 h), miraculin, at 1 mM, and mogroside V, at 100 µM, significantly reduced epithelial cell viability, an effect similar to saccharin, whereas stevia had no detrimental impact at any concentration. More worrying, at a longer time-point (48 h), miraculin and mogroside V caused significant epithelial cell death at 100 µM. At this concentration, miraculin and mogroside V also significantly elevated oxidative stress, whereas co-treatment with the pan anti-oxidant, NAC, abolished this effect, confirming ROS involvement in their activity. Similar antioxidant-mediated suppression of sweetener-induced ROS was noted with saccharin and has been reported previously^12,22^. At lower concentrations (10 µM), independent of cell death, miraculin and mogroside V disrupted epithelial barrier integrity, causing increased paracellular leakage and reduced TEER, while stevia preserved epithelial barrier homeostasis. These findings suggest that even natural sweeteners are not uniformly benign and have varying effects on intestinal function.

The intestinal epithelial barrier is central to maintaining selective permeability and immune tolerance. Increased epithelial cell death and disruption of tight junctions are known to drive barrier leakage, contributing to inflammation, infection, and dysbiosis^23,24^. Our results align with previous evidence showing that saccharin increases epithelial permeability^12^. Interestingly, both miraculin and mogroside V also impaired barrier integrity and decreased TEER through activation of the sweet taste receptor T1R3. Knockdown of T1R3 abrogated the permeability defects and loss of viability induced by these sweeteners via ROS production, confirming the role of T1R3 as a critical mediator of the epithelium. Stevia, on the other hand, did not cause leak and drop in TEER, and remained unchanged after the knockdown of T1R3. Miraculin and mogroside V are therefore acting through the sweet taste receptor to initiate a negative signalling response. The distinct effects observed may stem from variations in intracellular signalling pathways activated downstream of T1R3, potentially influenced by the structural differences among the natural sweeteners. These results are consistent with earlier studies suggesting that structural differences in artificial sweeteners modulate receptor binding and downstream signalling^25^. While mogroside V, structurally similar to sucrose, directly activates T1R3, miraculin’s action appears more complex, functioning as a pH-dependent taste modifier. As these features remained unaffected by T1R3 silencing with exposure to stevia, this suggest a protective or neutral role on barrier function^26^. However further studies with stevia and a barrier-disruptive agent, such as TNF-α, are needed to establish whether the effect is barrier-protective^27^.

To better understand the impact of natural sweeteners on intestinal permeability, gene expression analysis was performed. These studies revealed that miraculin and mogroside V significantly altered junctional gene expression, spanning tight, gap, and adherens junction components. Both miraculin and mogroside V upregulated tight junction genes (*CLDN2*, *CLDN3*, *CLDN7*, *CLDN10*) and gap junction genes (*GJA1*, *GJA3*, *GJA4*, *GJA5*), whilst also modulating adhesion-associated genes, *CAV1*, *ITGB3*, *DLL1*, and *DSG3*. Notably, *CLDN2* induction, as seen with miraculin and mogroside V, is a hallmark of leaky epithelia, frequently observed in inflammatory bowel disease (IBD), where it increases paracellular cation permeability and undermines barrier tightness^28^. Downregulation of *CLDN11* by miraculin and mogroside V may further destabilize adherens junction signalling, weakening epithelial cohesion^29^. Similarly, increases in *DLL1* mRNA with miraculin and mogroside V mirror findings by Ou et al. showing DLL1-medated Notch signalling is associated with a reduced colonic epithelial integrity^30^. Whilst the link between *GJA3* and *GJA4* and barrier function is not clear, outside of the epithelium these connexins have been shown to be upregulated in hypoxia or oxidative stress^31^. Therefore, the induction of ROS in response to miraculin and mogroside V exposure may elicit the increase in mRNA expression of *GJA3* and *GJA4*. However, the mRNA for the pro-sealant claudin, *CLDN3*, and pro-adhesive gap junction alpha, *GJA1* and *GJA5,* are also upregulated with miraculin and mogroside V exposure, suggesting that these sweeteners may stimulate a pan-junctional modulation signal rather than simply switching the epithelium to barrier disruption. Indeed, we previously observed that upregulation of claudin 3 rescued the epithelial barrier disruption induced by artificial sweeteners sucralose and aspartame^12^. As such, these mRNA may be upregulated as a compensatory mechanism and part of a wider disruption of adhesion-related genes in the intestinal epithelium. Stevia again showed a significantly different profile, maintaining baseline gene expression and preserving epithelial stability. Whilst this agrees with our findings that stevia has minimal effect on the intestinal epithelial cell line, as compared to miraculin and mogroside V, these findings differ from previous studies in the literature which show that stevia can upregulate barrier-protective proteins such as claudin-1, occludin, and ZO-1^26,32^. These differences may be due to these studies measuring protein versus mRNA expression or the concentration of stevia where we investigated 10 µM whilst Xu et al. exposed cells to 250 µM^26^. Changes of barrier function can induce harmful or beneficial effects on intestinal mucosa, depending on intestinal milieu. Increased epithelial permeability may lead to epithelial injury by allowing inappropriate influx of molecules through the TJ, which may induce immune activation. In some conditions, growth factors moved through TJ can bind and activate their basolateral receptors, activating proliferative signalling pathway. Alternatively, increased TJ permeability in the normal leaky epithelium can allow the wash out of molecules that may inappropriately diffuse from the intestinal lumen^33^. Thus, it is possible that stevia halts cell junction turnover to maintain a static epithelium which would be a negative for intestinal epithelial barrier turnover. However, our study only measured the gene expression of cell junction molecules therefore further study is needed to establish whether these changes translate to protein level and, more importantly, localisation, in the intestinal epithelial cell.

Taken together, these results suggest that miraculin and mogroside V, despite being natural, can negatively influence intestinal epithelial function in ways reminiscent of artificial sweeteners like saccharin, mediated primarily through T1R3-dependent pathways. Their induction of permeability, oxidative stress, and dysregulated junctional gene expression may predispose the epithelium to inflammation, altered ion transport, or pathological remodelling. Stevia, in contrast, demonstrates a protective role, supporting its status as a safer natural sweetener for gut health. Nonetheless, it is important to recognize contextual differences; while miraculin and mogroside V showed harmful effects in our model, other studies have reported beneficial roles, such as mogroside V’s ability to attenuate oxidative stress-induced intestinal damage in mice^34^ or miraculin’s nutritional benefits in malnourished cancer patients^35^. This highlights the complexity of sweetener biology, where concentration, context, and molecular structure dictate outcomes. Our findings, therefore, underscore the need for critical evaluation of natural sweeteners beyond their “healthy” perception. While stevia emerges as a relatively safe alternative, miraculin and mogroside V warrant careful assessment, particularly given their growing commercial use without established ADI values. Further in vivo and clinical studies are needed to clarify whether their epithelial-disruptive effects observed in vitro translate into adverse outcomes in humans, or whether compensatory mechanisms mitigate these risks.

## Supplementary Information

Supplementary Information.

## Data Availability

Data will be made available upon request to corresponding author.
