# Supplementary material for: The non-synthetic sweeteners, miraculin and mogroside V, but not stevia, disrupt the intestinal epithelial barrier function through a sweet taste receptor-dependent mechanism
Source: Sci Rep. 2025 Dec 29;15:44861. doi: 10.1038/s41598-025-28759-z (PMC12749673; doi:10.1038/s41598-025-28759-z)
Supplement: Supplementary file 1 — Supplementary Information. [file 41598_2025_28759_MOESM1_ESM.pptx]

## Slide 1
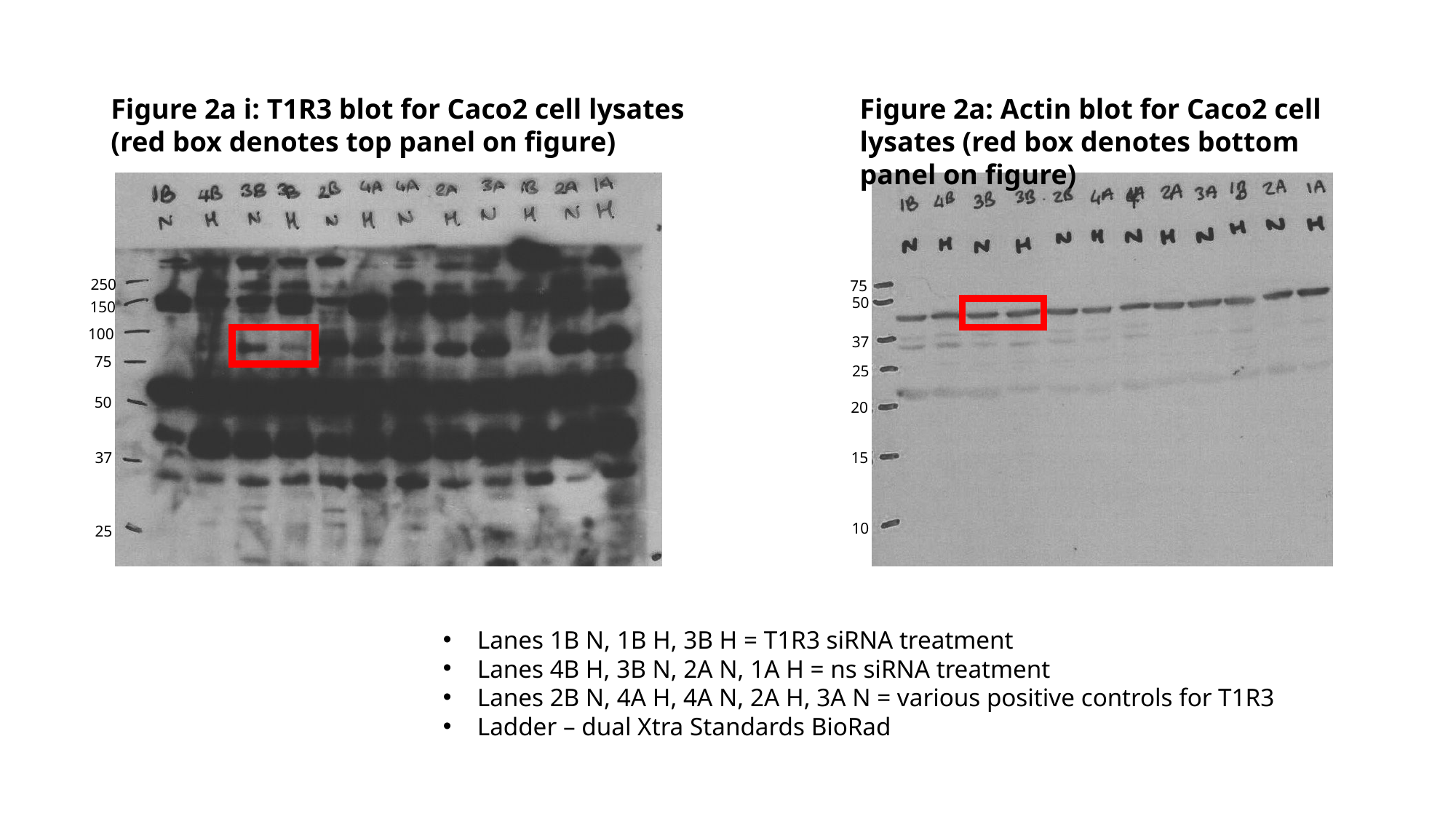

Figure 2a i: T1R3 blot for Caco2 cell lysates (red box denotes top panel on figure)
Figure 2a: Actin blot for Caco2 cell lysates (red box denotes bottom panel on figure)
250
150
100
75
50
37
25
75
50
37
25
20
15
10
Lanes 1B N, 1B H, 3B H = T1R3 siRNA treatment
Lanes 4B H, 3B N, 2A N, 1A H = ns siRNA treatment
Lanes 2B N, 4A H, 4A N, 2A H, 3A N = various positive controls for T1R3
Ladder – dual Xtra Standards BioRad
